# Expression of TSP50, SERCA2 and IL-8 in Colorectal Adenoma and Carcinoma: Correlation to Clinicopathological Factors

**DOI:** 10.3389/pore.2021.1609990

**Published:** 2021-10-21

**Authors:** Heba M. K. Youssef, Dina A. Radi, Marwa A. Abd El-Azeem

**Affiliations:** Pathology Department, Faculty of Medicine, Tanta University, Tanta, Egypt

**Keywords:** clinicopathological factors, colorectal adenoma, colorectal carcinoma, immunohistochemistry, IL-8, SERCA2 TSP50

## Abstract

**Background:** Colorectal cancer (CRC) is the third most common type of cancer, it is considered a genetically heterogeneous disease with different molecular pathways being involved in its initiation and progression. Testes-specific protease 50 (TSP50) gene is a member of cancer/testis antigens that encodes for threonine protease enzyme. Overexpression of TSP50 was found to enhance the progression and invasion of breast cancer and other malignant tumors. SERCA2 is widely expressed in several body tissues; its aberrant expression has been involved in many cancers. IL-8 is an inflammatory cytokine. Alongside its role in inflammation, its expression was reported to induce the migration of tumor cells.

**Aim:** Study the expression of TSP50, SERCA2 and IL-8 in colorectal adenoma (CRA), CRC and normal colonic tissues to compare the expression of these biomarkers in relation to clinicopathological parameters and prognostic factors.

**Results:** TSP50, SERCA2 and IL-8 expression varied between normal colonic tissues, CRA and CRC. Significant statistical association was detected between the three biomarkers’ overexpression and degree of dysplasia in CRA. Also, significant statistical relation was found between the three biomarkers’ overexpression and presence of lympho-vascular invasion, advanced TNM staging and high intra-tumoral inflammatory infiltrate. Multivariable analysis showed that the overexpression of the three biomarkers is significantly associated with worse prognosis.

**Conclusion:** The expression of TSP50, SERCA2 and IL-8 was different between the normal tissue and neoplastic colorectal tissue on one hand and between CRA and CRC on the other. Increased expression of these biomarkers in neoplastic epithelial cells of colorectal carcinoma is associated with adverse prognostic factors and could be considered as independent prognostic factors.

## Introduction

Colorectal cancer (CRC) is the third most common type of cancer, with a worldwide incidence of about 24.8% in 2020. In Egypt; CRC ranked tenth in males (5.8%) and ninth in females (6.2%) [1, 2]. It accounts for about 50% of all gastrointestinal tract-cancers with adenocarcinoma being the predominant histopathological type. Despite the continuous improvement in diagnosis and therapy, approximately 60% of colon cancer patients develop metastases [3].

Colorectal cancer is a genetically heterogeneous disease in which several and different molecular pathways are involved in transformation of normal colonic epithelium to malignant cells, tumor proliferation and invasion. The expanding knowledge of the molecular pathology of colon cancer resulted in classification of CRC into characteristic molecular subtypes based on gene expression in both tumor cells and infiltrating non tumorous stromal cells, to increase the accuracy of prognosis and design personalized therapies for better management of patients [4].

Testes-specific protease 50 (TSP50) gene, a member of cancer/testis antigens, encodes for a threonine protease enzyme which was first discovered in breast cancer cells. It is expressed in the normal spermatocytes of testes and is related to spermatogenesis [5]. Abnormal activation and overexpression of TSP50 was found to enhance the progression and invasion of breast cancer and other malignant tumors such as gastric [6], cervical [7] and lung [8] cancers. Recent studies reported that activation of TSP50 gene through DNA hypomethylation stimulates proliferation and transformation of cells while hypermethylation with subsequent TSP50 gene silencing or knocking down are associated with inhibition of cell proliferation, migration and inducing apoptosis [7]. As hypomethylation is common and prominent in CRC compared to normal colorectal tissue, TSP50 could be considered as a potential oncogene. In addition, TSP50 gene locus; 3p21.31 has been recognized as a susceptible locus for CRC in the Chinese population [9].

Disruption of calcium homeostasis becomes evident as an important step in the processes of tumorigenesis and metastasis through upregulation of genes’ expression which are involved in cellular proliferation, angiogenesis and invasion [10]. The level of intracellular Ca^2+^ is tightly regulated by Ca^2+^ channels and Ca^2+^ pumps which control the movement of Ca^2+^ across the plasma membrane and intracellular storage organelles [11]. One of the Ca^2+^ pumps belonging to the family of P-Type ATPases is Sarco/Endoplasmic Reticulum Ca^2+^-ATPase or SERCA. SERCA is responsible for segregating calcium in the endoplasmic reticulum (ER), the richest store for intracellular calcium [12]. There are 3 genes coding the SERCA1- 3 pumps, with different expression levels and tissue distributions in the body, one of these three isoforms is SERCA2 which is widely expressed in several body tissues [13]. Aberrant expression of SERCA2 either up-or downregulation have been involved in many cancers such as lung and prostatic cancers mirroring the link between disturbances in Ca^2+^ homeostasis on one hand and cancer initiation and progression on the other hand [14]. However the exact role of SERCA2 expression in CRC and its relation to clinicopathological factors have not been cleared up.

Colorectal carcinoma has been found to express a variety of chemokines, including the multifunctional cytokine interleukin-8 (IL-8), a member of the CXC chemokine family of inflammatory cytokines that stimulates the migration of distinct subsets of cells [15]. IL-8 and other inflammatory cytokines have been shown to act as potent chemo-attractants for leucocytes, such as neutrophils and natural killer cells [16]. In recent years, IL-8 was reported to induce the migration of tumor cells and its expression was correlated with tumor growth, angiogenesis and metastatic potential in various human carcinomas [17-19].

The expression of TSP50 as a potential oncoprotein in association with SERCA2 and inflammatory cytokine (IL-8) in colorectal adenoma (CRA) and CRC and the relation of these biomarkers’ expression to intratumoral inflammatory infiltrate and other clinicopathological and prognostic factors in CRC have not been clarified. Thus, the aim of this work is to study the expression of TSP50, SERCA2 and IL-8 in CRA and CRC to find the relation of these biomarkers’ expression to intratumoral inflammatory infiltrate and other clinicopathological and prognostic factors.

## Materials and Methods

After the approval of local ethical committee, Faculty of Medicine, Tanta University (34340/12/20), 107 selected specimens of CRA and primary CRC (29 CRA and 78 CRC) along with 12 normal colonic mucosal tissues obtained from non-specific colonic lesions, were retrospectively collected from the archive of pathology department and private labs. The specimens were either colonoscopic biopsies (for CRA) or colectomies (for CRC). None of colorectal carcinoma cases had received neoadjuvant chemotherapy.

H&E stained sections were revised to confirm the diagnosis and to assess the grade of dysplasia of CRA, the degree of tumor differentiation, lympho-vascular invasion (LVI) and the intra-tumoral inflammatory cell infiltrate score of CRC. The dysplasia of CRA was graded according to a two-tier grading system into a low and high-grade dysplasia (LGD and HGD respectively) [20]. Colorectal carcinomas were graded into three grades; well, moderately and poorly differentiated [21] and they were staged according to TNM staging system [22]. The intra-tumoral inflammatory cellular infiltrate [lymphocytes (ITL) and neutrophils (ITN)] of CRC was evaluated and scored as follow: score 0; no increase in inflammatory cells, score 1; a mild increase in inflammatory cells without destruction of the cancer cells, score 2; a moderate increase in inflammatory cells with some destruction of the cancer cells, and score 3; severe inflammatory reaction with frequent destruction of the cancer cell islet. Specimens were further grouped into either low-inflammatory group and high-inflammatory group. Tumors with scores 0 and 1 were categorized as low-inflammatory, while those with scores 2 and 3 were categorized as high-inflammatory group [23].

For immunohistochemistry, paraffin blocks of embedded tissues were cut into 5-μm-thick sections, deparaffinized in two changes xylene, rehydrated in two changes of alcohol then rinsed in distilled water. Antigen retrieval was performed by first preheating the staining dish containing citrate buffer (10 mM citric acid, 0.05% Tween20, pH 6.0) until temperature reaches 95–100°C then sections were incubated in it for 20–40 min. Sections were incubated overnight at 4°C, to increase the affinity of the antibodies used to their antigens, with the following primary antibodies: anti-TSP50 polyclonal (1:25; ABclonal, United States), anti-SERCA2 polyoclonal (1:100; ABclonal, United States) and anti-IL-8 polyclonal (1:100; ABclonal, United States). The slides were then counter stained with hematoxylin. Breast carcinoma was used as a positive control for TSP50. For IL-8 and SERCA2, infiltrating neutrophils were used as an internal positive control.

### Immunohistochemical Assessment

Immunohistochemical evaluation of TSP50 protein cytoplasmic staining was performed using semi-quantitative scoring method by multiplying the intensity of staining and the percentage of positive tumor cells. For staining intensity, the sections were scored as 0 (negative), 1 (mild), 2 (moderate) and 3 (strong), and for the percentages: 0 (0–10%), 1 (10–30%), 2 (31–50%), 3 (51–70%) and 4 (71–100%). The final scores were graded as follows: − (0); + (1–3); ++ (4–8); +++ (9–12). The sections were classified into two groups: low expression (−,+); and high expression (++,+++) for statistical analysis [24].

For SERCA2, the specimens were considered positive if >5% of the colonic epithelial cells showed cytoplasmic and/or plasma membrane staining. Semiquantitative scores were used for SERCA2 stains according to the percentage of positively stained cells (+1: <25%; +2: 25–50%; +3: 51–75%; and +4: >75%). Tumors showing expression scores of ++, +++, or ++++ were considered as the high expression group, whereas those with scores of + or negative staining were regarded as the low expression group [25].

Cytoplasmic and membranous staining for IL-8 were evaluated. The intensity of staining was scored as 0 (negative), 1 (weak), 2 (moderate), or 3 (strong). The percentage of stained cells was grouped as: no staining = 0, 1–10% of stained cells = 1, 11–50% = 2, 51–80% = 3, and 81–100% = 4. Values of <4 and ≥4 divide the patients into low and high IL-8 expression groups respectively [26].

### Statistical Analysis

Statistical analysis was conducted using SPSS (version 20) (Chicago, IL, United States). The association between clinicopathological factors and the expression of biomarkers was performed using Chi‐square (*x* ^2^) test. Correlation between biomarkers’ expression was performed using Spearman correlation (r) and regression analysis was used for multivariable analysis of prognostic factors. The results were considered as statistically significant if the *p* value was <0.05.

## Results

### Clinicopathological Characteristics of CRA Cases

For the 29 CRA cases, the age of patients ranged from 40–62 years with a mean age of (42.3 ± 2.08). Eighteen (62.1%) cases were males and the other eleven (37.9%) cases were females. Twenty four adenomas were located in the colon (16 in the left side and 8 in the right side) and five adenomas (17.2%) in the rectum. Thirteen (44.8%) adenomas were of tubular type and the remaining 16 (55.2%) showed tubulovillous configuration. LGD was detected in 15 (51.7%) and HGD in 14 (48.3%) CRA cases Table 1.

**TABLE 1:** Clinicopathologic characteristics of CRA cases.

Factor	CRA n (%)
Age	
Mean ± SD	42.3 ± 2.08
<45	6 (20.7)
≥45	23 (79.3)
Sex	
Male	18 (62.1)
Female	11 (37.9)
Anatomical site	
Colon	
Right side	8 (27.6)
Left side	16 (55.2)
Rectum	5 (17.2)
Histological type	
Tubular	13 (44.8)
Tubulovillous	16 (55.2)
Dysplasia	
LGD	15 (51.7)
HGD	14 (48.3)

LGD, low-grade dysplasia; HGD, high-grade dysplasia.

### Clinicopathological Characteristics of CRC Cases

A total of 78 CRC cases were studied, the age of patients was ranged from 42–72 years with a mean age of (60.4 ± 7.41), 48 (61.5%) cases were males and 30 (38.5%) cases were females. Twenty (25.6%) tumors were located in the right colon, 46 (59%) tumors were in the left colon and 12 (15.4%) tumors were in the rectum. Most of the cases were adenocarcinoma NOS type (64.1%). Moderate and poor degrees of differentiation were detected in 38 (48.7%) and 22 (28.2%) carcinomas respectively, while well differentiated carcinomas were found in 18 (23.1%) specimens. LVI was found in 45 tumors (57.7%). For TNM staging, T_1_ tumors were identified in 11 (14.1%) cases, T_2_ in 53 (68%) cases and T_3_ in 14 (17.9%) cases. The regional lymph node invasion was recognized in 65 cases (83.3%) and distant metastasis was found in 30 (38.5%) cases. For intra-tumoral inflammation, high ITL and ITN scores were encountered in 40 (51.3%) and 38 (48.7%) tumors respectively Table 2.

**TABLE 2:** Clinicopathological characteristics of CRC cases.

Clinicopathologic characteristics	n (%)
Age	
Mean ± SD	60.4 ± 7.41
<45	18 (23.1)
≥45	60 (76.9)
Sex	
Male	48 (61.5)
Female	30 (38.5)
Anatomical site	
Colon	
Right side	20 (25.6)
Left side	46 (59)
Rectum	12 (15.4)
Histological type	
Adenocarcinoma NOS	50 (64.1)
Mucinous	18 (23.1)
Signet ring	10 (12.8)
Grade	
I	18 (23.1)
II	38 (48.7)
III	22 (28.2)
LVI	
Absent	33 (42.3)
Present	45 (57.7)
TNM Staging	
T	
T_1_	11 (14.1)
T_2_	53 (68)
T_3_	14 (17.9)
N	
N_0_	13 (16.7)
N_1_	49 (62.8)
N_2_	16 (20.5)
M	
M_0_	48 (61.5)
M_1_	30 (38.5)
ITL	
Low	34 (43.6)
High	44 (56.4)
ITN	
Low	40 (51.3)
High	38 (48.7)

LVI, lympho-vascular invasion; T, primary tumor size; N, regional lymph node spread; M, distant metastasis; ITL, intra-tumoral lymphocytes; ITN, intra-tumoral neutrophils.

### Expression of TSP50, SERCA2 and IL-8 in Neoplastic Cells of CRA

**FIGURE 1:**
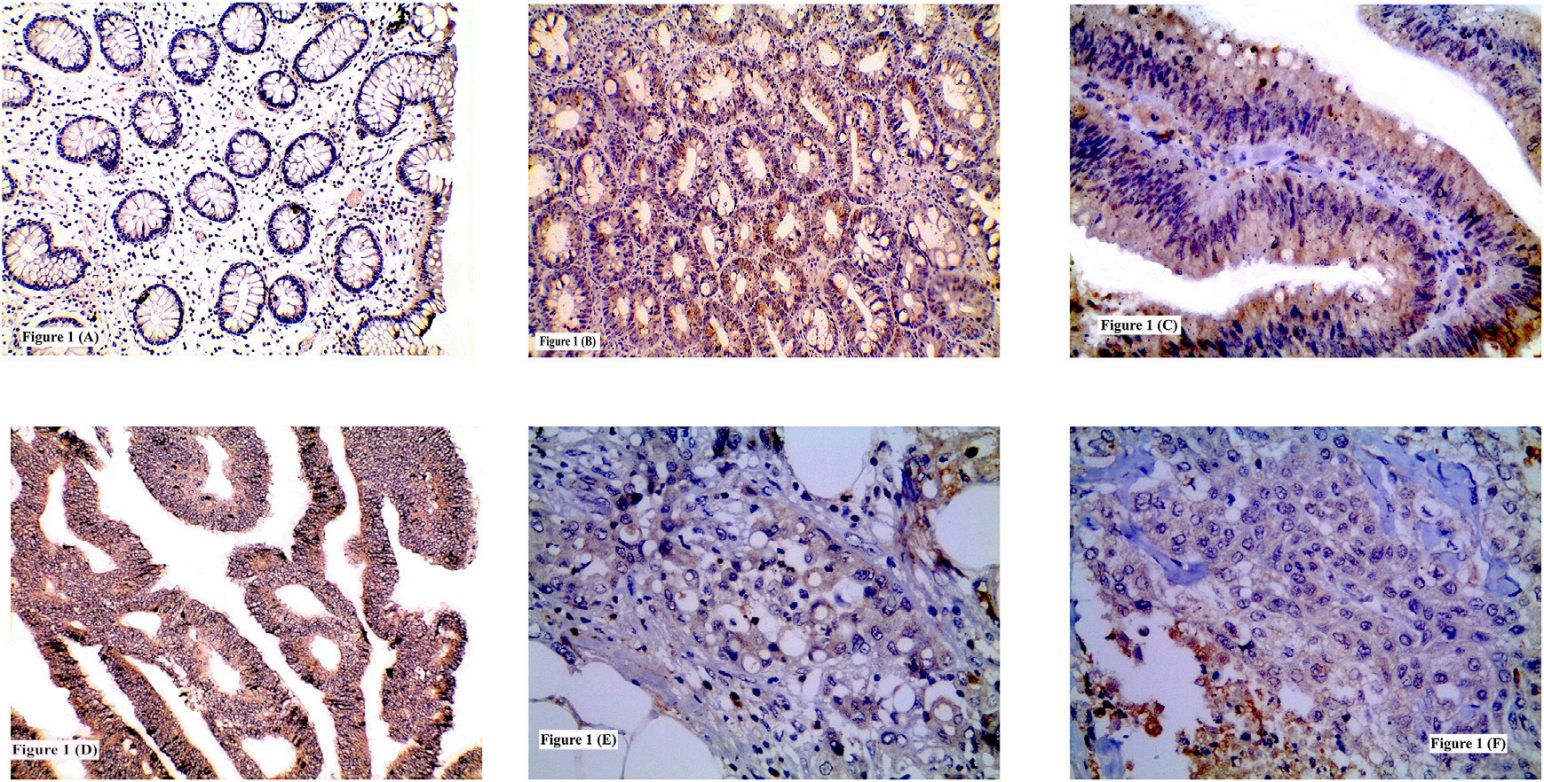
Immunohistochemical expression of TSP50 showing: **(A)** Low expression in normal colonic tissue; **(B)** High expression in tubular adenoma; **(C)** High expression in tubulovillous adenoma with high grade dysplasia; **(D)** High expression in grade II CRC; **(E)** High expression in signet ring CRC; **(F)** Low expression in grade III CRC. (**A**, **B**, **D** ×200) (**C**, **E**, **F** ×400).

High TSP50 expression was observed in 15 adenomas and it was significantly identified more in CRAs with tubulovillous architecture (68.8%) compared to tubular ones (30.8%) and in adenomas HGD (71.4%) than adenomas with LGD (33.3%) (*p* = 0.042 and 0.040 respectively). No significant statistical relation was found between TSP50 immunostaining and CRA location (*p* = 0.564) Table 3 and Figures 1B,C.

**TABLE 3:** Expression of TSP50, SERCA2 and IL-8 in CRA

Factor	TSP50			SERCA2			IL-8		
	Low *n* = 14	High *n* = 15	*x* ^*2*^/*p* value	Low *n* = 16	High *n* = 13	*x* ^*2*^/*p* value	Low *n* = 19	High *n* = 10	*x* ^*2*^/*p* value
Histological type									
Tubular	9 (69.2)	4 (30.8)	4.144/0.042*	8 (61.5)	5 (38.5)	0.388/0.534	10 (77)	3 (23)	1.357/0.244
Tubulovillous	5 (31.3)	11 (68.8)		8 (50)	8 (50)		9 (56.3)	7 (43.7)	
Location									
Colonic	11 (45.8)	13 (54.2)	0.333/0.564	12 (50)	12 (50)	1.506/0.220	15 (62.5)	9 (37.5)	0.561/0.454
Rectal	3 (60)	2 (40)		4 (80)	1 (20)		4 (80)	1 (20)	
Dysplasia									
LGD	10 (66.7)	5 (33.3)	4.209/0.040*	11 (73.3)	4 (26.7)	4.144	13 (86.7)	2 (13.3)	6.152/0.013*
HGD	4 (28.6)	10 (71.4)		5 (35.7)	9 (64.3)	0.042*	6 (42.9)	8 (57.1)	

*p value < 0.05.

LGD, low grade dysplasia; HGD, high grade dysplasia; CRA, colorectal adenoma.

**FIGURE 2:**
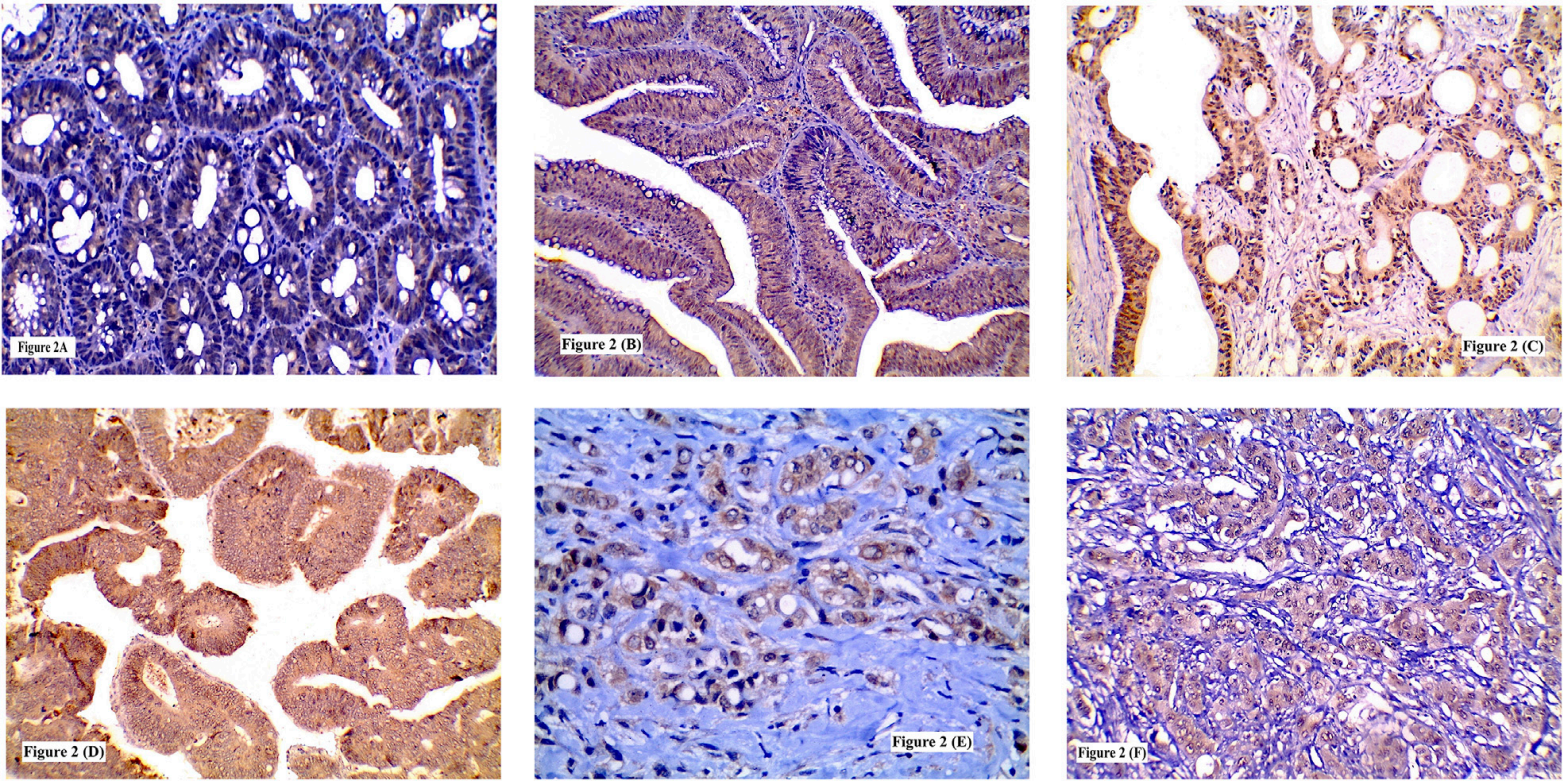
Immunohistochemical expression of SERCA2 showing: **(A)** Low expression in low grade tubular adenoma; **(B)** High expression in high grade tubulovillous adenoma; **(C)** High expression in grade I CRC; **(D)** High expression in grade II CRC; **(E)** High expression in signet ring CRC; **(F)** High expression in grade III CRC. (**A**, **B**, **C**, **D**, **F** ×200) (**E** ×400).

Higher SERCA2 immunolabelling was detected in tubulovillous (50%) more than tubular adenomas (38.5%) and in CRAs with HGD more than those with LGD (64.3 and 26.7% respectively). Significant statistical relation was recognized between high SERCA2 expression and the degree of dysplasia (*p* = 0.042). No significant statistical difference was found between SERCA2 expression and CRA histologic type or location (*p* = 0.534 and 0.220 respectively) Table 3 and Figures 2A,B.

**FIGURE 3:**
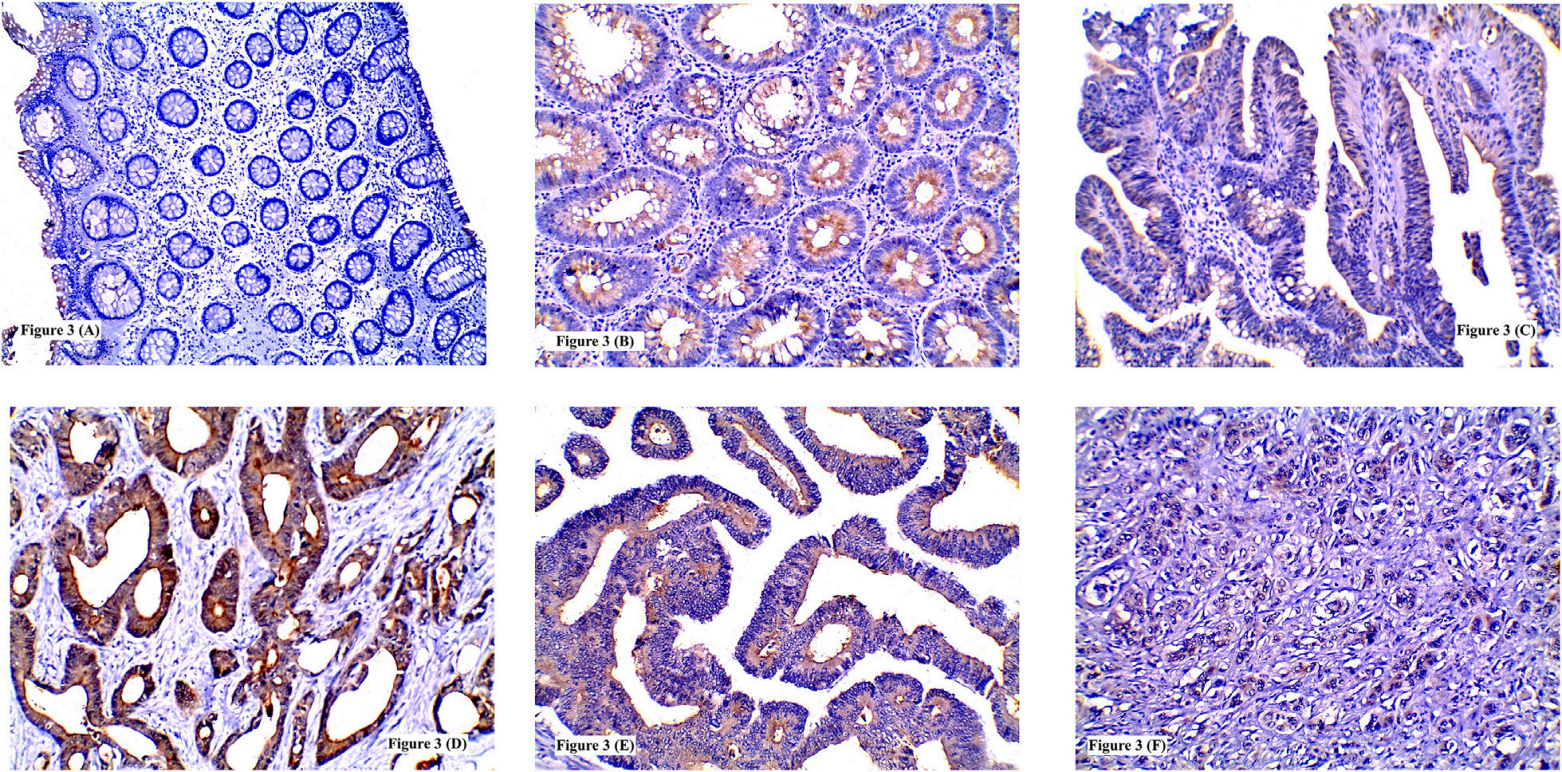
Immunohistochemical expression of IL-8 showing: **(A)** Low expression in normal colonic tissue; **(B)** High expression in tubular adenoma; **(C)** Low expression in tubulovillous adenoma with low grade dysplasia; **(D)** High expression in grade I CRC; **(E)** High expression in grade II CRC; **(F)** High expression in poorly differentiated CRC. (**A**, **F** ×100) (**B**, **C**, **D**, **E** ×200).

IL-8 high expression was recognized in 10 CRA whereas low expression was detected in 19 CRA. No significant statistical relation was found between IL-8 expression and adenoma type or location (*p* = 0.244 and 0.454 respectively). High IL-8 immunostaining was significantly detected more frequently in CRA with HGD (57.1%) than LGD (13.3%) (*p* = 0.013) Table 3 and Figures 3B,C.

### Expression of TSP50, SERCA2 and IL-8 in the Neoplastic Cells of CRC

High TSP50 expression was found in 47/78 cases and it was significantly associated with the presence of LVI (73.3%, *p* = 0.006), advanced TNM staging (T stage; *p* = 0.008, N stage; *p* = 0.0001 and M stage; *p* = 0.0001) and high intra-tumoral inflammatory scores whether lymphocytic (75%, *p* = 0.002) or neutrophilic (76.3%, *p* = 0.005). TSP50 protein overexpression was demonstrated in 55.6, 71 and 45.5% of grade I, II and III tumors respectively. Although poorly differentiated tumors showed high TSP50 expression less frequently than well and moderately differentiated tumors in neoplastic cells, however non-neoplastic stromal cells of grade III tumors exhibited high TSP50 protein expression. No statistically significant relationship was detected between TSP50 immunostaining and tumor location (*p* = 0.882), histological type (*p* = 0.073) or grade (*p* = 0.133) Table 4 and Figures 1D–F.

**TABLE 4:** Expression of TSP50, SERCA2 and IL-8 in neoplastic cells of CRC.

Factor	TSP50			SERCA2			IL-8		
	Low *n* = 31 *n* (%)	High *n* = 47 *n* (%)	*x* ^*2*^/*p* value	Low *n* = 37 *n* (%)	High *n* = 41 *n* (%)	*x* ^*2*^/*p* value	Low *n* = 34 *n* (%)	High *n* = 44 *n* (%)	*x* ^*2*^/*p* value
Anatomical site									
Colon									
Right side	12 (60)	8 (40)	0.022/0.882	9 (45)	11 (55)	1.131/0.288	6 (30)	14 (70)	0.021/0.884
Left side	14 (30.4)	32 (69.6)		24 (52.2)	22 (47.2)		23 (50)	23 (50)	
Rectum	5 (41.7)	7 (58.3)		4 (33.3)	8 (66.7)		5 (41.7)	7 (58.3)	
Histological type									
Adenocarcinoma NOS	16 (32)	34 (68)	5.241/0.073	23 (46)	27 (54)	0.739/0.691	17 (34)	33 (66)	5.213/0.074
Mucinous	8 (44.4)	10 (55.6)		10 (55.6)	8 (44.4)		11 (61.1)	7 (38.9)	
Signet ring	7 (70)	3 (30)		4 (40)	6 (60)		6 (60)	4 (40)	
Grade									
I	8 (44.4)	10 (55.6)	4.028/0.133	13 (72.2)	5 (27.7)	8.022/0.018*	14 (77.8)	4 (22.2)	11.253/0.004*
II	11 (29)	27 (71)		18 (47.4)	20 (52.6)		12 (31.6)	26 (68.4)	
III	12 (54.5)	10 (45.5)		6 (27.3)	16 (72.7)		8 (36.4)	14 (63.6)	
LVI									
Absent	19 (57.6)	14 (42.4)	7.595/0.006*	21 (63.6)	12 (36.4)	6.021/0.014*	23 (69.7)	10 (30.3)	15.855/0.0001*
Present	12 (26.7)	33 (73.3)		16 (35.6)	29 (64.4)		11 (24.4)	34 (75.6)	
TNM Staging									
T									
T_1_	9 (81.8)	2 (18.2)	9.601/0.008*	10 (90.9)	1 (9.1)	12.234/0.002*	8 (72.7)	3 (27.3)	8.749/0.013*
T_2_	18 (34)	35 (66)		24 (45.3)	29 (54.7)		24 (45.3)	29 (54.7)	
T_3_	4 (28.6)	10 (71.4)		3 (21.4)	11 (78.6)		2 (14.3)	12 (85.7)	
N									
N_0_	12 (92.3)	1 (7.7)	18.157/0.0001*	10 (77)	3 (23)	9.860/0.007*	11 (84.6)	2 (15.4)	11.609/0.003*
N_1_	15 (30.6)	34 (69.4)		24 (49)	25 (51)		19 (38.8)	30 (61.2)	
N_2_	4 (25)	12 (75)		3 (18.8)	13 (81.2)		4 (25)	12 (75)	
M									
M_0_	28 (58.3)	20 (41.7)	18.009/0.0001*	30 (62.5)	18 (37.5)	11.358/0.001*	26 (54.2)	22 (45.8)	5.678/0.017*
M_1_	3 (10)	27 (90)		7 (23.3)	23 (76.7)		8 (26.7)	22 (73.3)	
ITL									
Low	20 (58.8)	14 (41.2)	9.162/0.002*	19 (55.9)	15 (44.1)	1.725/0.189	22 (64.7)	12 (35.3)	10.930/0.001*
High	11 (25)	33 (75)		18 (41)	26 (59)		12 (27.3)	32 (72.7)	
ITN									
Low	22 (55)	18 (45)	7.908/0.005*	29 (72.5)	11 (27.5)	20.686/0.0001*	24 (60)	16 (40)	8.992/0.003*
High	9 (23.7)	29 (76.3)		8 (21.1)	30 (78.9)		10 (26.3)	28 (73.7)	

*p value < 0.05.

LVI, lympho-vascular invasion; T, primary tumor size; N, regional lymph node spread; M, distant metastasis; ITL, intra-tumoral lymphocytes; ITN, intra-tumoral neutrophils.

SERCA2 cytoplasmic overexpression in neoplastic cells was identified in only 5 grade I tumors (27.7%), most of the grade II tumors (52.6%) and in the majority of grade III tumors (72.7%). High SERCA2 expression was found more frequently among tumors with LVI (64.4%) than those without (36.4%). Moreover, SERCA2 overexpression was linked with advanced TNM staging. Tumors with high ITN infiltration (78.9%) showed higher SERCA2 protein expression than those with low ITN infiltration (27.5%) or ITL (either low-or high, 44.1 and 59% respectively) infiltration. Significant statistical association was revealed between high SERCA2 protein expression and tumor grade (*p* = 0.018), LVI (*p* = 0.014), TNM staging (*p* = 0.002, 0.007 and 0.001 respectively) and high ITN infiltration (*p* = 0.0001). No significant relation was found between SERCA2 immunolabelling and tumor location (*p* = 0.288), histological type (*p* = 0.691) or ITL infiltration (*p* = 0.189) Table 4 and Figures 2C–F.

High IL-8 immunoreactivity was identified in 44/78 tumors. This high IL-5 protein expression was significantly detected in moderately differentiated (68.4%) and poorly differentiated (63.6%) carcinomas compared to well differentiated ones (22.2%) (*p* = 0.004). Also, tumors with LVI (75.6%) showed significant higher IL-8 expression more than those without LVI (30.3%) (*p* = 0.0001). Similarly, increased IL-8 expression was significantly linked to increase in tumor size (85.7%) (*p* = 0.013) advanced N staging (N_2_ = 75%) (*p* = 0.003) and presence of distant metastasis (73.3%) (*p* = 0.017). Tumors with high ITL and/or ITN exhibited significant high IL-8 protein immunolabeling (72.7 and 73.7% respectively) (*p* = 0.001 and 0.003 respectively). No significant statistical association was found between IL-8 immunoreactivity and tumor site (*p* = 0.884) or histological type (*p* = 0.074) Table 4 and Figures 3D–F.

Multivariable analysis of independent prognostic factors: anatomical site, histological type, tumor grade, LVI (absent versus present), TN staging, intra-tumoral inflammatory infiltrate (low versus high), expression of TSP50, SERCA2 and IL-8 proteins (low expression versus high) was conducted showing that TSP50, SERCA2 and IL-8 overexpression were significantly associated with adverse prognosis along with high tumor grade, LVI, advanced T stage, presence of regional lymph node invasion and high ITL Table 5.

**TABLE 5:** Multivariable analysis of factors affecting prognosis in colorectal carcinoma cases.

	Metastasis		Multivariable *p* value
Anatomical site			
Colon			
Right side	7/20	35%	0.451
Left side	17/46	36.9%	
Rectum	6/12	50%	
Histological type			
Adenocarcinoma NOS	20/50	40%	0.656
Mucinous	4/18	22.2%	
Signet ring	6/10	60%	
Grade			
I	0/18	0%	<0.0001*
II	18/38	47.4%	
III	12/22	54.5%	
LVI			
Absent	6/33	18.2%	0.001*
Present	24/45	53.3%	
T			
T_1_	0/11	0%	0.004*
T_2_	22/53	41.5%	
T_3_	8/14	57.1%	
N			
N_0_	0/13	0%	0.025*
N_1_	23/49	46.9%	
N_2_	7/16	43.8%	
ITL			
Low	11/34	32.4%	0.026*
High	19/44	43.2	
ITN			
Low	16/40	40%	0.778
High	14/38	36.8%	
TSP50			
Low	3/31	9.7%	<0.0001*
High	27/47	51.1%	
SERCA2			
Low	7/37	18.9%	0.001*
High	23/41	56.1%	
IL-8			
Low	8/34	23.5%	0.017*
High	22/44	50%	

*p value < 0.05.

LVI, lympho-vascular invasion; T, primary tumor size; N, regional lymph node spread; ITL, intra-tumoral lymphocytes; ITN, intra-tumoral neutrophils.

### Correlation Between TSP50, SERCA2 and IL-8 Expression in the Neoplastic Cells of CRC

A significant positive correlation was found between TSP50 expression on one side and SERCA2 and IL-8 expression on the other side. As 32 (78%) and 37 (84.1%) tumors of high SERCA2 and IL-8 immunoreactivity respectively showed TSP50 overexpression as well (r = 0.383 and 0.554, *p* = 0.001 and <0.0001 respectively). Furthermore, A significant statistical positive correlation was detected between SERCA2 and IL-8 expression, in which 33 (75%) tumors with high IL-8 immunostaining showed high SERCA2 expression too (r = 0.511, *p* < 0.0001) Table 6.

**TABLE 6:** Correlation between TSP50, SERCA2 and IL-8 expression in the neoplastic cells of CRC.

	TSP50		r	*p* Value	SERCA2		r	*p* Value
	Low n (%)	High n (%)			Low n (%)	High n (%)		
SERCA2								
Low	22 (59.5)	15 (40.5)	0.383	0.001*	-	-	-	-
High	9 (22)	32 (78)			-	-	-	-
IL-8								
Low	24 (70.6)	10 (29.4)	0.554	<0.0001*	26 (76.4)	8 (23.5)	0.511	<0.0001*
High	7 (15.9)	37 (84.1)			11 (25)	33 (75)		

*p value < 0.05.

r, correlation.

### Comparing the Expression of TSP50, SERCA2 and IL-8 in Normal Tissues, CRA and CRC

TSP50 expression was variable, being low in colorectal normal epithelium (Figure 1A), low-to- high in CRA and mostly high in CRC. TSP50 expression scores in CRC were significantly higher than those in colorectal normal tissues or CRA (*p* = 0.001). SERCA2 immunoreactivity also varied between normal epithelium, CRA CRC cases. Significant increase in SERCA2 expression was observed in CRC (*p* = 0.003). Similarly, IL-8 protein was significantly higher in CRCs compared to normal epithelium specimens (Figure 3A) and CRA (*p* < 0.0001) Table 7.

**TABLE 7:** Expression of TSP50, SERCA2 and IL-8 in normal tissues, CRA and CRC cases.

Factor	TSP50			SERCA2			IL-8		
	Low	High	x^2^/*p* value	Low	High	x^2^/*p* value	Low	High	x^2^/*p* value
Normal	12 (100)	0 (0)	15.133/0.001*	12 (100)	0 (0)	11.598/0.003*	12 (100)	0 (0)	15.718/<0.0001*
CRA	14 (48.3)	15 (51.7)		16 (55.2)	13 (44.8)		19 (65.5)	10 (34.5)	
CRC	31 (39.7)	47 (60.3		37 (47.4)	41 (52.6)		34 (43.6)	44 (56.4)	

*p value < 0.05.

CRA, colorectal adenoma; CRC, colorectal carcinoma.

## Discussion

Colorectal carcinoma still remains a challenging disease due to several contributing factors: a variety of molecular transformation pathways other than the traditional APC, KRAS and tumor suppressor gene p53 mutations are implicated in CRC development [27], altered biochemical cellular metabolism of neoplastic cells, molecular heterogeneity between the primary CRC and its metastasis and the mysterious role of intra-tumoral inflammatory cellular infiltrate. All these multifactor could result in dissimilarities between the biological behavior of malignant cells regarding the manner of growth, invasion and metastasis. Moreover, this heterogeneity will reflect on the response of patients to therapy.

Therefore, the aim of this study is to investigate the roles of TSP50 as an oncoprotein with altered calcium metabolism and inflammatory cytokine namely SERCA2 and IL-8 respectively in CRA and CRC, understanding their roles in CRC initiation and propagation and the relation of these biomarkers’ expression to the intratumoral inflammatory infiltrate and other clinicopathological and prognostic factors.

As far as we know, this is the first study that correlates the immunohistochemical detection of TSP50, SERCA2 and IL-8 in CRA and CRC with clinicopathological factors.

Testes-specific protease 50 (TSP50) is a unique member of cancer/testis antigens, with exception of testes, it is not expressed in normal tissues. It is activated and re-expressed in human cancers such as breast cancer and other cancers [28].

In the present study the expression of TSP50 was examined in CRA, CRC as well as in normal colonic mucosa. It was found that TSP50 was not detected or weakly expressed in the normal mucosa tissues. Whereas TSP50 expression scores were significantly varied between CRA and CRC lesions. The significant increase of TSP50 expression found in CRC and CRA compared to normal mucosal tissues is of great value for considering TSP50 as a crucial protein for the process of tumorigenesis.

Also, the TSP50 expression in CRA neoplastic cells was tested in relation to clinicopathological parameters. It was found that high TSP50 expression was significantly identified in adenomas with tubulovillous architecture more than tubular adenomas and in adenomas with HGD more than those with LGD.

Regarding the TSP50 expression in CRC malignant cells and its relation to clinicopathological and prognostic factors, TSP50 overexpression in epithelial cells was observed more abundantly in moderately differentiated CRC than poorly differentiated tumors with no significant association between high TSP50 expression and tumor grade. On the other hand, TSP50 overexpression in CRC was significantly associated with advanced TNM staging and presence of LVI. Additionally, TSP50 protein upregulation was shown to be significantly associated with high intra-tumoral inflammatory infiltrate (both lymphocytes and neutrophils).

The findings of the present work supported the role of TSP50 in tumor cellular proliferation, migration and invasion reported by previous study which found that TSP50 mutation has led to diminished tumorigenic effect of TSP50 with subsequent decrease in *in vitro* cellular proliferation in nude mice [7]. Moreover, TSP50 epithelial overexpression was found to enhance the proliferation and invasion of cervical cancer cells *via* its protease activity [29]. Similarly, other study showed that TSP50 overexpression was significantly associated with poor prognostic outcomes in non-small cell lung cancer compared to those with low TSP50 expression [8]. Besides, high expression of TSP50 in neoplastic epithelial cells was associated with advanced malignancy and poor prognosis in human gastric cancer and colorectal carcinoma, respectively [24,30].

The interesting finding in the present work was the paradoxical low TSP50 epithelial expression in 54.5% of poorly differentiated CRC including the signet ring cell type and its high expression in associated non-neoplastic stromal cells; which may be attributed to the dual role that TSP50 plays in both epithelial neoplastic cells and stromal cells. Its epithelial expression enhances tumor cellular proliferation and migration, while the stromal expression of TSP50 facilitates the neoplastic epithelial cell invasion through its threonine protease activity. This could explain the heterogeneity of TSP50 epithelial and stromal expression in high grade CRC in the current work. Also, TSP50 expression was found to be variable depending on tumor type, genesis and microenvironment [31]. Along with the fact that malignant cells may alter or modify the expression of proteins that regulate cancer cell growth and invasion through epigenetic DNA hyper or hypomethylation or genetic mutations could add another point of explanation [32].

Intracellular Ca^2+^ is kept in a state of equilibrium and regulated by Ca^2+^ signaling and Ca^2+^ channels. Disruption of this equilibrium stimulates the expression of proteins that can lead to cellular transformation, proliferation, invasion and metastasis. One of the regulators of intracellular Ca^2+^ signaling is SERCA2 [13].

SERCA2 expression in CRA, CRC and normal mucosal tissues were investigated in the present study. It was observed that SERCA2 expression was variable between normal and neoplastic lesions (CRA and CRC) with significant increase in its expression in neoplastic lesions as none of the normal colonic tissues showed high SERCA2 expression. This was in agreement with previous study which found that the mean expression level of SERCA2 mRNA in malignant colorectal tissues was significantly higher than that in noncancerous tissues [25].

Furtherly, the SERCA2 expression in CRA neoplastic cells in relation to clinicopathological parameters was studied. It was found that high SERCA2 expression was significantly associated with adenoma with HGD than adenoma with LGD. No significant relation was detected between SERCA2 expression and histological architecture of adenoma.

Besides the fact that the expression of SERCA2 in CRC epithelial cells was tested, it was found that SERCA2 overexpression was significantly related to high tumor grade, advanced TNM staging, presence of LVI and high ITN (but not lymphocytic) infiltrate. These findings were similar to previous studies which showed that colorectal carcinoma with increased SERCA2 expression was significantly associated with serosal invasion, lymph node metastasis and advanced tumor stage compared to those with low SERCA2 expression meaning that SERCA2 may be a crucial biomarker in the development and progression of CRC [25, 33, 34].

In addition, previous studies reported the key role of intracellular calcium remodeling in colorectal cancer process that includes cellular proliferation, migration, invasion and metastasis [34, 35]. As during cellular transformation, disturbances of intracellular Ca^2+^ occur due to altered expression of Ca^2+^ channel proteins which control the process of Ca^2+^ pump [36]. One of these Ca^2+^ channel proteins is SERCA2 which is important for ER Ca^2+^ stores refill to maintain efficient protein folding and maturation, whereas overexpression causing ER Ca^2+^ stores overload that leads to ER stress observed in various cancers [37].

Our results supported the predominant association of SERCA2 overexpression with poor prognostic factors of CRC, as SERCA2 overexpression in cancer cells is a prerequisite for evasion of apoptosis causing depletion of intracellular Ca^2+^ *via* increases Ca^2+^ influx into the ER with subsequent inactivation of caspases proved by previous study which showed that HCT-116 human colon cancer cells apoptosis can be triggered through increasing intracellular Ca^2+^ either by increasing extracellular Ca^2+^ influx or ER Ca^2+^ efflux [38], besides it enhances cellular proliferation, motility and invasion *via* the activation of mitogen-activated protein kinase (MAPK) signal transduction pathway through SERCA2 induced-ER stress which are essential for colorectal carcinogenesis [34, 39].

Furthermore, inhibition of SERCA2 expression was proved to induce G_2_/M cycle arrest and growth elimination in SW480 human colon adenocarcinoma epithelial cells *in vitro* and *in vivo* [40].

IL-8 is a multifunctional chemokine which was found to induce tumor growth, angiogenesis and metastatic potential in CRC.

IL-8 expression was examined in CRA, CRC as well as in normal colonic mucosa. A significant increase in IL-8 expression rate was detected in CRC more than in CRA and normal colonic mucosa. Similarly, previous studies found that IL-8 is upregulated in CRC compared to CRA and normal colonic mucosa [41, 42].

Subsequently, IL-8 expression was studied in CRA in relation to clinicopathological parameters, where no significant relations between IL-8 overexpression and CRA type or location were found, while high IL-8 immunostaining was significantly associated with CRA showing HGD. A Similar finding in other study which found that IL-8 mRNA level was significantly elevated with increased degree of dysplasia in the adenomas [42].

In addition, IL-8 expression in neoplastic cells of CRC was investigated, and it was detected that IL-8 protein overexpression was significantly linked to high tumor grade, presence of LVI, increased tumor size, regional lymph node, distant metastasis and high intra-tumoral inflammatory infiltrate. No significant relation was detected between IL-8 expression and tumor site or histological type.

Parallel to our results, previous studies showed that IL-8 expression is significantly upregulated in CRC neoplastic cells and this upregulation was associated with increased tumor growth, invasion and distant metastasis [43–46]. Added to that, human and mice *in vitro* colon cancer cells grew rapidly in IL-8 rich microenvironment with enhanced angiogenesis compared to normal microenvironment [26]. Moreover, IL-8 and its receptors promoted primary CRC progression and liver metastasis by direct effect on tumor cells, angiogenesis stimulation or regulating the tumor stroma, they also reported that high IL-8 levels in the tissues were significantly related to tumor grade, stage, lymph node and liver metastasis [47].

The growth signaling pathway (MAPK) is stimulated by interaction of IL-8 and its receptors that may be expressed in tumor associated stromal cells particularly the inflammatory cells [48], this explains the significant increase of IL-8 expression in relation to high intra-tumoral inflammatory infiltrate detected in the present study.

It is noteworthy that, in the current study, high expression of TSP50, SERCA2 and IL-8 were significantly associated with high intra-tumoral inflammatory infiltrate in CRC. This finding supports the idea of the role of inflammatory microenvironment in increasing mutation rates and enhancing the division of mutated cells. This may be due to release of reactive oxygen species (ROS) from activated inflammatory cells which are able to promote DNA mutation and genomic instability in the epithelial cells [49].

Regarding the correlation between the expression of TSP50, SERCA2 and IL-8 in CRC, significant positive correlation was found between the three biomarkers’ expression in neoplastic epithelial cells. This finding could be explained by the fact that both inflammatory process and tumorigenesis are related to disruption of Ca^2+^ homeostasis which is involved in DNA and gene methylation [36]. TSP50 gene abnormal methylation and the subsequent TSP50 protein expression in tumorous epithelial and stromal cells may be related to SERCA2 expression and intracellular Ca^2+^ alteration. Moreover, alteration of intracellular Ca^2+^ stimulates the secretion of pro-inflammatory cytokines from epithelial neoplastic cells as well as tumor associated stromal cells as a result of protein unfolding [50]. In addition to that, both SERCA2 and IL-8 have synergistic action through stimulating the MAPK growth signaling pathway in tumor cells [39, 48]. Thus the increased expression of IL-8 and SERCA2 in relation to high intratumoral inflammatory infiltrate with subsequent increase in TSP50 protein expression in CRC, in the present study, and the significant association of these biomarkers’ expression to adverse clinicopathological and prognostic factors namely: LVI and advanced TNM staging are of crucial value for understanding the role of these biomarkers in CRC initiation, growth and invasion.

## Conclusion

Based on our findings. The aberrant expression of TSP50, SERCA2 and IL-8 in CRA and CRC compared to normal colonic mucosa could represent a different molecular transformation pathway. Also, increased expression of these biomarkers in neoplastic epithelial cells of CRC is associated with adverse prognostic factors through facilitating several hallmarks of cancer and could be considered as independent prognostic factors. Therefore, combination immunotherapy targeting TSP50, SERCA2 and IL-8 molecules would be a valuable effective therapy for inhibiting colorectal carcinoma initiation, proliferation and metastasis.

## Data Availability

The original contributions presented in the study are included in the article/supplementary material, further inquiries can be directed to the corresponding author.
